# A pilot study of optical neuronavigation‐guided brain biopsy in the horse using anatomic landmarks and fiducial arrays for patient registration

**DOI:** 10.1111/jvim.15813

**Published:** 2020-05-29

**Authors:** Lawrence Santistevan, Jeremiah Easley, Audrey Ruple, Sam Monck, Elissa Randall, Fred Wininger, Rebecca A. Packer

**Affiliations:** ^1^ Department of Clinical Sciences, College of Veterinary Medicine and Biomedical Sciences Colorado State University Fort Collins Colorado USA; ^2^ Department of Public Health, College of Health and Human Sciences Purdue University West Lafayette Indiana USA; ^3^ Department of Environmental and Radiological Health Sciences, College of Veterinary Medicine and Biomedical Sciences Colorado State University Fort Collins Colorado USA; ^4^ Charlotte Animal Referral and Emergency Charlotte North Carolina USA

**Keywords:** equine, intracranial, intraoperative guidance, natural landmark, neurosurgery, nonfiducial

## Abstract

**Background:**

Optical neuronavigation‐guided intracranial surgery has become increasingly common in veterinary medicine, but its use has not yet been described in horses.

**Objectives:**

To determine the feasibility of optical neuronavigation‐guided intracranial biopsy procedures in the horse, compare the use of the standard fiducial array and anatomic landmarks for patient registration, and evaluate surgeon experience.

**Animals:**

Six equine cadaver heads.

**Methods:**

Computed tomography images of each specimen were acquired, with the fiducial array rigidly secured to the frontal bone. Six targets were selected in each specimen. Patient registration was performed separately for 3 targets using the fiducial array, and for 3 targets using anatomic landmarks. In lieu of biopsy, 1 mm diameter wire seeds were placed at each target. Postoperative images were coregistered with the planning scan to calculate Euclidian distance from the tip of the seed to the target.

**Results:**

No statistical difference between registration techniques was identified. The impact of surgeon experience was examined for each technique using a Mann‐Whitney *U* test. The experienced surgeon was significantly closer to the intended target (median = 2.52 mm) than were the novice surgeons (median = 6.55 mm) using the fiducial array (*P* = .001). Although not statistically significant (*P* = .31), for the experienced surgeon the median distance to target was similar when registering with the fiducial array (2.47 mm) and anatomic landmarks (2.58 mm).

**Conclusions and Clinical Importance:**

Registration using both fiducial arrays and anatomic landmarks for brain biopsy using optical neuronavigation in horses is feasible.

**Abbreviations**
CT: computed tomography
MRI: magnetic resonance imaging
PET: positron emission tomography

## 1 INTRODUCTION

Optical neuronavigation‐guided intracranial surgery is the standard of care in human medicine, and is becoming more common in veterinary medicine.^1^, ^2^, ^3^, ^4^ Although veterinary‐specific navigation and biopsy systems exist, their design is derived from systems used in humans, and is not optimized for use in equids.^1^, ^4^, ^5^, ^6^, ^7^, ^8^, ^9^ Although these systems are accurate,^1^, ^2^, ^3^, ^4^ anatomic differences among species make some aspects of these systems logistically difficult in veterinary patients. In order to acquire planning images, general anesthesia is required to securely fasten fiducial markers to the patient. Furthermore, the imaging must either be performed a priori during the diagnostic scan, not knowing if the fiducial arrays are necessary, or a second anesthesia and imaging session is required for planning after the initial diagnostic scans. These planning sessions add logistical complexity for the clinician, and the need for additional anesthesia and recovery events can be dangerous for the horse.^10^, ^11^, ^12^ With regard to optical neuronavigation in humans, the recent emergence of anatomic landmarks and facial contouring could be applied to veterinary medicine.^13^, ^14^ By using anatomic landmarks as patient registration, the need for fiducial arrays and a separate anesthesia and planning session could be avoided if they show the similar accuracies as in current procedures used in humans.

The Brainsight Vet 2 neuronavigation system (Rogue Research, Inc, Montreal, Canada) is a veterinary‐specific system and is extensively used in small animal and research settings for optical navigation. Clinical applications in dogs and cats have been validated using this system,^1^, ^2^, ^4^ as well as in research applications for nonhuman primates^15^, ^16^, ^17^, ^18^ and sheep.^19^, ^20^, ^21^ The system is compatible with computed tomography (CT)‐guided and magnetic resonance imaging (MRI)‐guided procedures, both of which are becoming more common in equine medicine. Furthermore, intracranial biopsy or resection is emerging as a relevant treatment modality for large animal species with granulomas, abscesses, and pituitary tumors, and neuronavigation would provide valuable guidance during these procedures.^22^, ^23^

Our purpose was to assess feasibility of using the Brainsight optical neuronavigation system in horses using CT‐guided neuronavigation, compare 2 registration protocols (standard frontal fiducial array and anatomic landmarks for patient registration to the navigation system), and compare the outcomes of surgeons with and without experience in neuronavigation procedures.

## 2 METHODS

### 2.1 Animals

The study used cadavers (n = 6), of horses euthanized for reasons unrelated to the study. All specimens were approved for educational use in accordance with appropriate Institutional Care and Use Committee or Clinical Review Board protocols, permissions, or exemptions. Cadavers were derived from 6 adult horses of unknown size and age. Cadavers were decapitated at the atlanto‐occipital junction after euthanasia and cadaver heads were stored frozen until use. Once thawed and the procedure commenced, no further storage or freeze‐thaw cycles were performed so as to minimize variation in brain shift or pneumocephalus between procedural components.

### 2.2 Image acquisition

**FIGURE 1:**
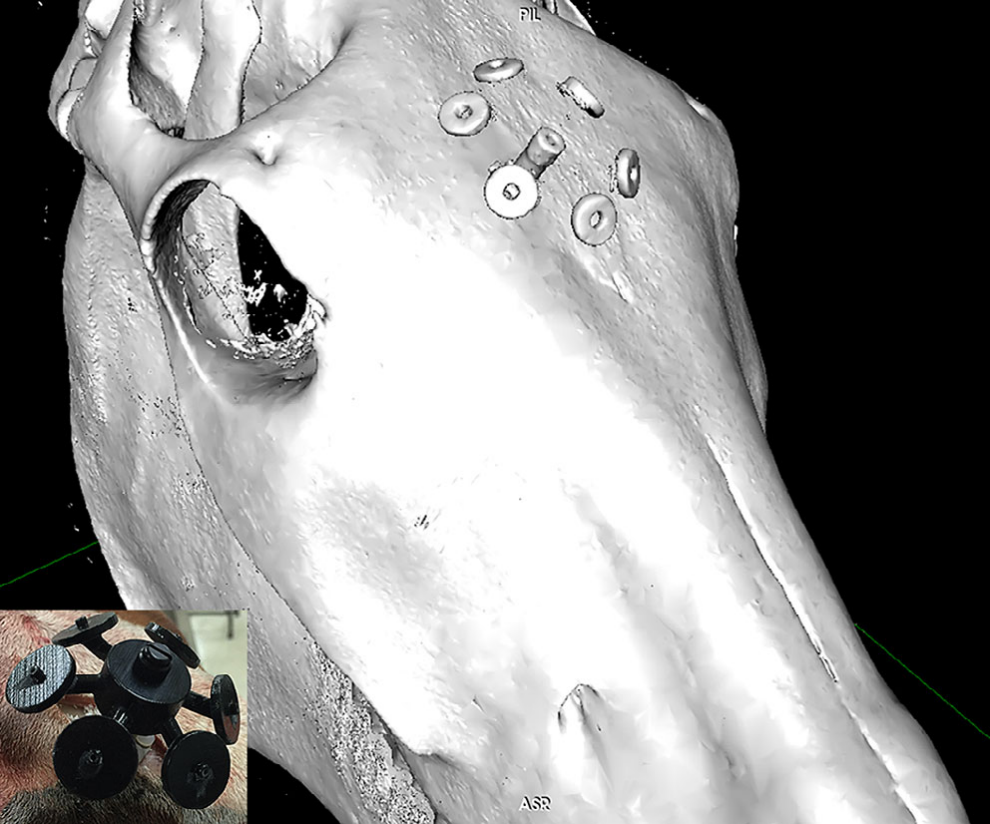
Computed tomographic reconstruction of a horse skull with the fiducial array secured to the frontal bone for patient registration in the Brainsight neuronavigation system. The array (inset) can be attached or detached from a low profile base plate, such that the skin can be opposed over the baseplate if imaging and surgery are performed on separate days

A fiducial array was rigidly secured to the frontal bone of each specimen using M3 × 5 mm ceramic screws, predrilled with a 2.36 mm drill bit. Each fiducial array had 6 markers in the array (Figure 1). This array is securely attached to the frontal bone, but the apparatus may be dislodged with moderate force. The cadaver heads were placed in dorsal recumbency and CT was performed on each equine head for neuronavigation planning. The CT images were acquired using a Philips positron emission tomography (PET) CT hybrid (16‐slice CT, 4‐slice PET), with a matrix of 768 × 768, voxel size of 0.8 mm × 0.8 mm × 1.0 mm (1.0 mm slice thickness with no gap or overlap), collimation of 16 × 0.75 mm, and pitch of 0.688.

### 2.3 Surgical planning

The images were imported into the neuronavigation system and surgical planning was performed using Brainsight Vet 2.0 (Rogue Research, Inc). Three‐dimensional (3D) reconstruction images of the skull and skin surfaces then were generated by the Brainsight system to prepare each specimen for surgical planning and registration for fiducial arrays and anatomic landmarks. To evaluate differences in accuracy between the 2 methods of registration (standard fiducial array and anatomic landmarks), 6 targets were randomly created using the Brainsight system to simulate the act of targeting a tumor biopsy or injection site, 3 for each registration method. Targets were placed in 6 consistent locations: rostral, middle, and caudal cerebrum, bilaterally. Rostral targets were located in the rostral parietal lobe, middle targets were located in the caudal parietal or temporal lobes, and caudal targets were located in the occipital lobe. All targets were placed at a minimum depth of 1.5 cm within brain tissue. A random number chart was used to determine the pattern of targets used and with which registration method in each specimen. Fiducial arrays (n = 6) and anatomic landmarks (n ≥ 7) were registered on the 3D reconstructions of the skull and skin. According to system requirements, a minimum of 6 landmarks should be used for navigation. Anatomic landmarks included left and right medial canthus, left and right lateral canthus, left and right supraorbital foramen, left and right infraorbital foramen, left and right facial crest point, and bregma (Table S1). The infraorbital foramen initially was used as a registration point bilaterally, but repeatable identification of a specific point on that structure was difficult to achieve. Consequently, the study design was modified and the facial crest was substituted for the infraorbital foramen for cases 3 to 6. Additionally, the left eye of horse 3 was distorted, and thus those points were omitted from registration in that case.

### 2.4 Biopsy procedure

**FIGURE 2:**
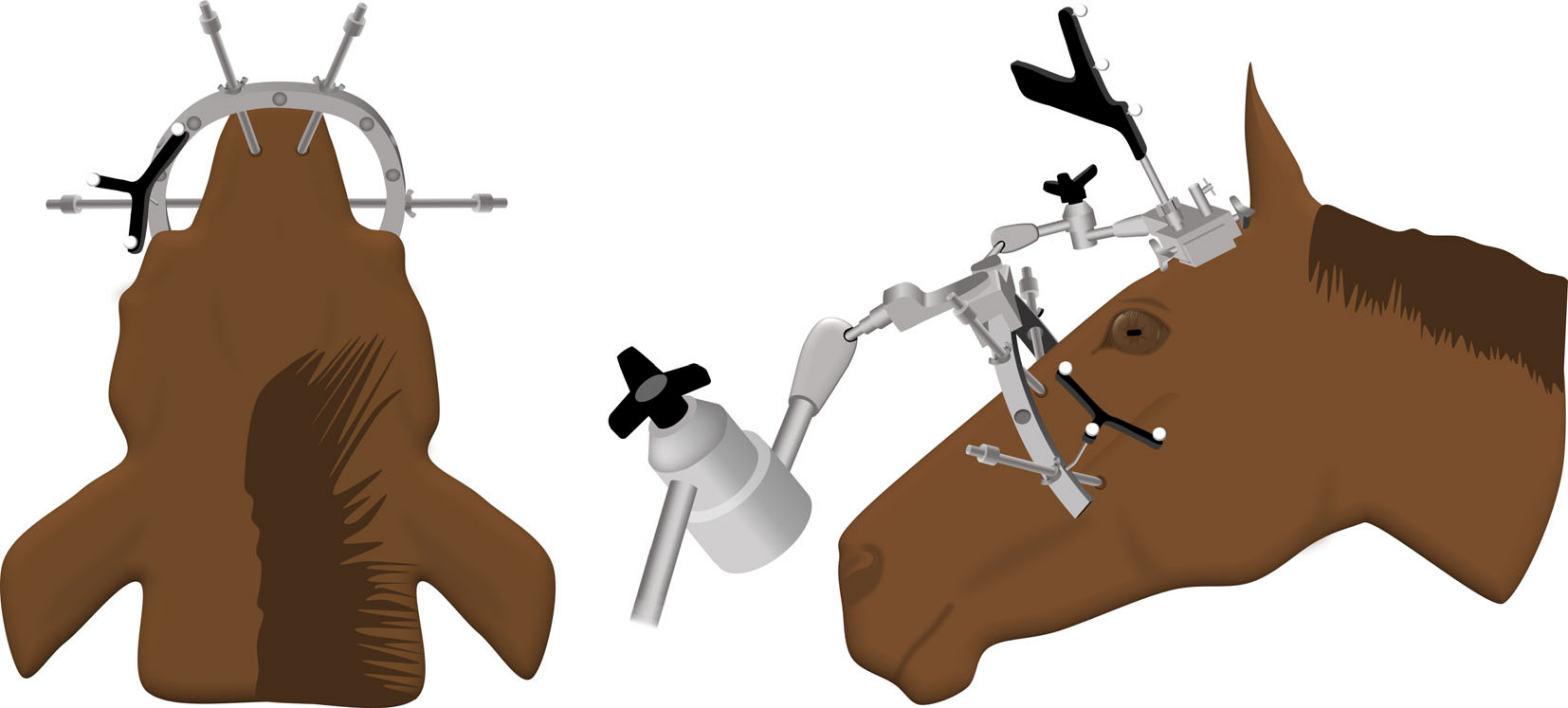
Illustration of the caudal and lateral perspectives depicting the placement of the halo as secured to the equine head. Four pins converged to secure the halo. Pins were directed such that they securely rested on the left and right nasal bones, and the left and right maxillary bones just below the rostral aspect of the facial crest

**FIGURE 3:**
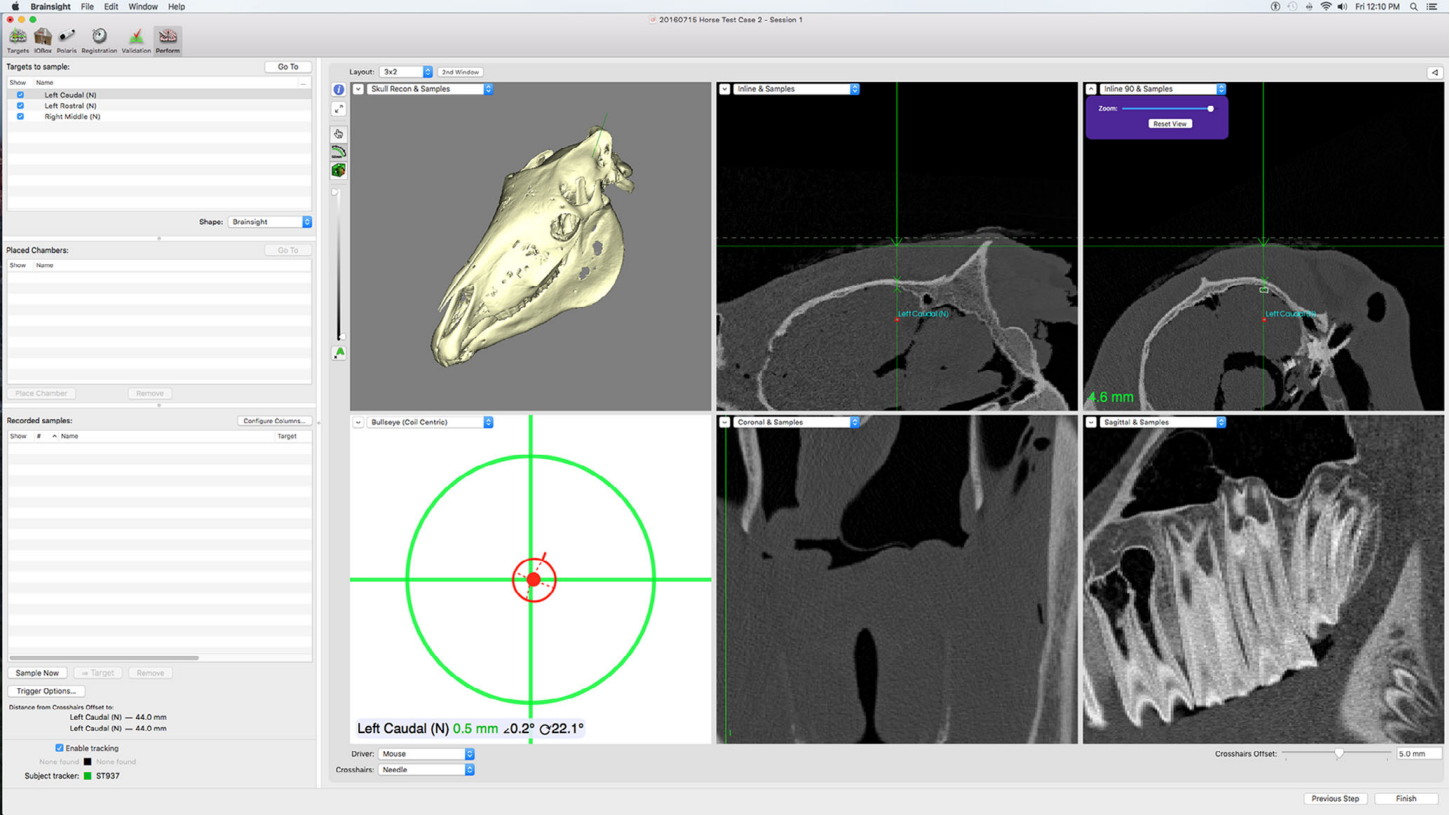
Screenshot of active neuronavigation using the Brainsight system. Note that the lower left of the image indicates that that the surgeon is on target (red dot aligned with green crosshairs) and on trajectory (red circle centered on green crosshairs). If the surgeon is off target, the red dot is not centered. If the surgeon is off trajectory, the red circle shows a red cone coming off the green crosshairs, indicating offset. The remaining 5 image tiles can be set according to surgeon preference for various static CT image planes, or active inline or perpendicular (called inline‐90) planes. In this figure, the top middle and top right images show the inline and inline‐90 images, respectively, as well as the intended targets (red point). The fine green line in these same two images indicates the position of the biopsy needle. In inline and inline‐90 images, the green line representing the biopsy needle remains stationary and the images move to align with the trajectory of the needle. In the static CT image planes (not shown), the images remain stationary and the fine green lines moves to indicate the needle trajectory. In this way, the surgeon can visualize biopsy position. CT, computed tomography

After surgical planning and registration, the biopsy procedures were performed according to standard procedure.^1^ Procedures were performed or supervised by either an experienced surgeon (a board‐certified veterinary neurologist and neurosurgeon with 14 years of experience in neurosurgery and 8 years of experience using the Brainsight Vet 2 neuronavigation system during routine intracranial procedures in dogs) or a novice surgeon (a board‐certified equine surgeon with 8 years of experience in the field of equine surgery but without previous experience using neuronavigation systems). The experienced surgeon performed the biopsies for the first 12 targets, and the novice surgeon performed the biopsies for the remaining 24 targets. A veterinary student without prior experience with neuronavigation systems was involved in all procedures. Each head was secured in the halo with 4 pins, with the patient tracking array attached to the halo to provide the link to the neuronavigation system. Skin incisions (1 cm) must be made to allow the pins to penetrate tissues and rest securely against the skull. Pin location can vary provided that all 4 pins converge to secure the head. In this study, 2 pins originated from the lateral aspect of the head and were directed craniomedially to rest on the left and right maxillary bones just below the rostral aspect of the facial crest. Two additional pins originated from the dorsal aspect of the head and were directed ventrally to rest on the left and right nasal bones (Figure 2). Specific adaptations of the system were not required, but because of the larger size of the horse relative to the dog, the halo was attached such that the opening of the C‐shaped halo was applied rostro‐caudally, with the opening directed caudally (which is the opposite position of that used in the dog). The arm that holds the halo cannot support the entire weight of an equine head, but with the head resting on a table, the halo provided secure attachment. For consistency, the halo and array were not moved during the entire procedure, including acquisition of data from both registration methods. Registration and validation of the anatomic or fiducial points were performed according to standard procedures for the system. Biopsy procedures followed those recommended for the Brainsight system. A 1 to 2 cm skin incision was made with a #10 scalpel blade over the site of entry for each planned biopsy target and trajectory. As is typical with neuronavigation systems, the system includes instrumentation for targeting to the intended biopsy site and trajectory, and performing a burr hole and durotomy over the entry site. Once the targeting was complete and the needle‐guide apparatus was aligned (Figure 3), the guide was secured in position. The burr hole was created through the needle‐guide apparatus, and drilling occurred using a hexagonal drill bit included with the system. A depth stop is included in the system, and facilitated drilling to the appropriate depth to penetrate bone, and helped prevent damage to brain tissue. A double‐pronged tissue pick, also included with the system, was used to penetrate the dura at the burr hole site. Procedures for needle entry to the targeted trajectory and depth to target were followed, and included setting the needle depth guide to the appropriate depth to target, and inserting the needle accordingly. In lieu of biopsy, however, 1 mm diameter × 5 mm long brass wire seeds were implanted. The system includes 2 biopsy needles, 1 side‐cutting needle, and an open‐ended aspiration cannula. This open‐ended cannula was used to implant the wire seeds. These brass seeds were implanted with the intent that the distal tip of the seed would be at the site of our intended targets by using the trajectory pathways previously (preoperatively) planned using Brainsight. After all 6 targets were implanted with the wire seeds, the specimen was scanned postoperatively using the same CT scanner and protocol that was used for presurgical planning. This process was repeated for each of the 6 cadaver heads.

### 2.5 Image analysis and coregistration

**FIGURE 4:**
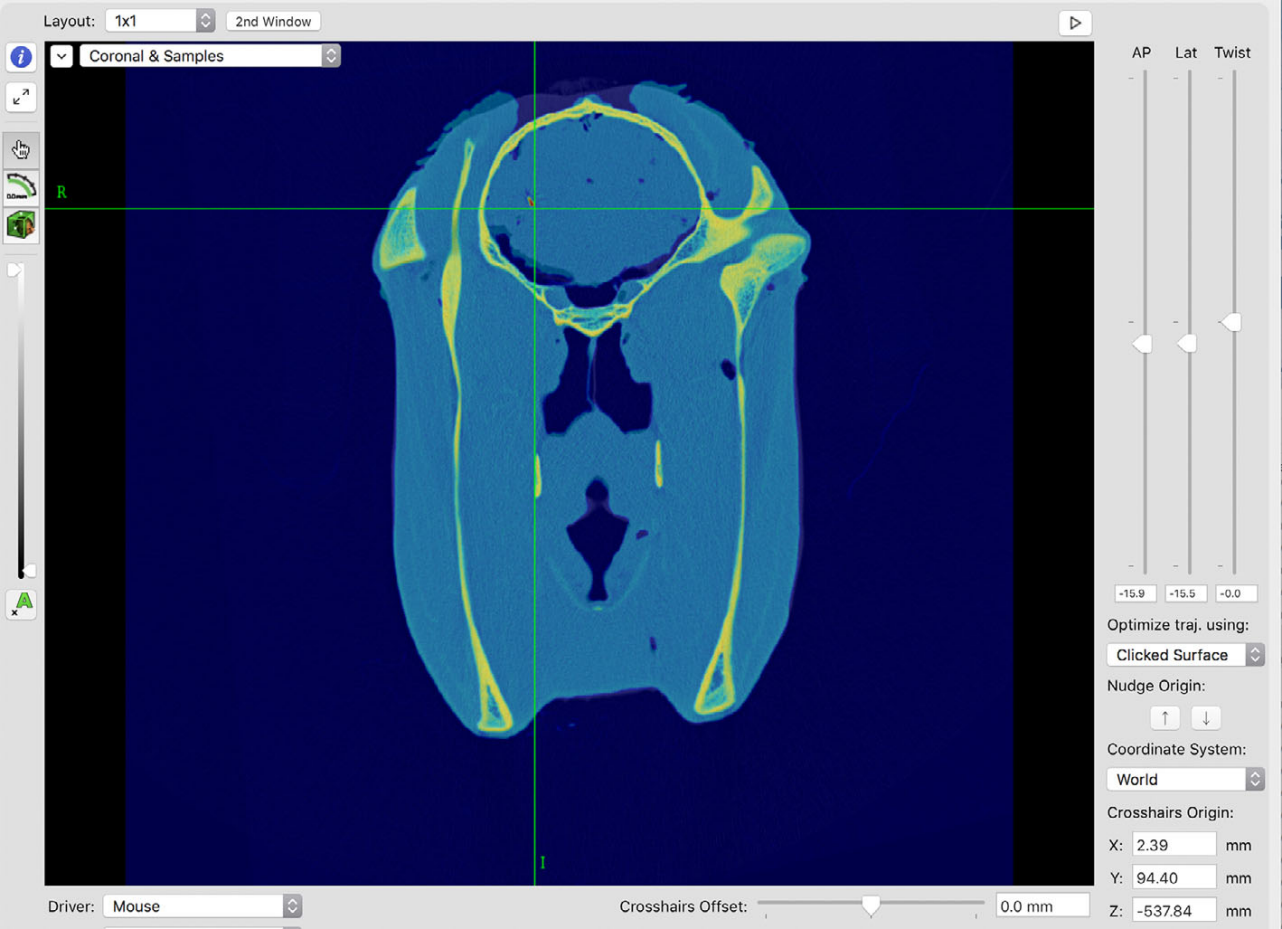
Coregistered images showing the position of the brass seed (red/yellow scaled color). The green crosshairs indicate the location of the seed tip. The coordinates of this position are shown in the lower right corner of the image, and were used to calculate the distance from the intended target

Preoperative and postoperative CT scans were coregistered in Intellispace Portal (version 8 Lot 8.0.1.20640, Phillips Medical Systems, Netherlands) and then imported back into Brainsight to calculate the distance from the seed tip to the intended target. The location of the seed tip was marked on the coregistered images, and the Brainsight system then generated the coordinates of the seed tip in the x, y, and z planes (Figure 4). These coordinates were compared to the coordinates of the intended target, and Euclidian distance from the intended target (*d*) to the actual location of the seed tip (*d*′) was calculated using the following formula: Difference (*d*, *d*′) =  √ [(*x* − *x*′)^2^ + (*y* − *y*′)^2^ + (*z* − *z*′)^2^] where *x*, *y*, and *z* represent the coordinates of the intended target and *x*′, *y*′, and *z*′ represent the coordinates of the actual location. An optional rotational plane (available for electrode implantation where aspect is relevant) was not clinically relevant to performing biopsies and was not used in the study.

### 2.6 Statistical analyses

Data were tested for normality using the Shapiro‐Wilk test. Differences between anatomical and fiducial registration methods were compared using a Wilcoxon matched‐pairs signed rank test. Differences based upon surgical experience level were compared between investigators using a Mann‐Whitney *U* test. The significance threshold was set at *P* < .05.

## 3 RESULTS

The Brainsight Vet 2 neuronavigation system was compatible for use with the equine skull using both fiducial arrays and anatomic landmarks. Some cadavers were distorted as a result of freeze‐thaw cycles and noncompliance of the skin and muscles of the head, such that individual anatomic landmarks could not be used and had to be omitted. The specific anatomic landmarks used for each specimen are provided in Table S1.

Descriptive data and comparative results from anatomic and fiducial registration are presented in Table 1. The median deviation from the targets was 3.76 mm and 5.75 mm for anatomic landmarks and fiducial arrays, respectively. No statistical difference between the anatomic and fiducial array registration techniques was identified (*P* = .52). The impact of surgeon experience was examined for all targets, as well as separately for each technique. The surgeon experienced in optical neuronavigation procedures was significantly closer to the intended target (median = 2.52 mm; interquartile range [IQR] = 1.36; SD = 3.45) than the novice surgeon (median = 6.55 mm; IQR = 6.63; SD = 5.37) (*P* = .001). When evaluating the specific registration techniques separately, the experienced surgeon was significantly closer to the intended target (median = 2.47 mm; IQR = 2.30; SD = 1.54) than was the novice surgeon (median = 8.62 mm; IQR = 6.45; SD = 3.37) using the fiducial array (*P* = .001), but not anatomic landmarks (*P* = .12; Table 1). Although not statistically significant (*P* = .31), for the experienced surgeon the median distance from target was similar when registering with the fiducial array (2.47 mm) and anatomic landmarks (2.58 mm; Table 1). Our data did not show evidence of sequential improvement for either surgeon, based on evaluation of descriptive statistics for individual horses over time.

**TABLE 1:** Descriptive data of each methodologic approach as compared to each other and when controlling for experience of surgeon

Approach	Surgeon experience	Median distance from target (mm)	IQR	SD	95% CI
Fiducial (N = 18)		5.75	7.28	3.88	3.09‐10.15
	Experienced^*^ (N = 6)	2.47	2.30	1.54	1.43‐5.64
	Novice^*^ (N = 12)	8.62	6.45	3.37	5.04‐12.06
Anatomic (N = 18)		3.76	4.79	6.26	2.77‐6.86
	Experienced (N = 6)	2.58	3.86	4.71	2.00‐14.06
	Novice (N = 12)	5.17	5.65	6.93	3.03‐9.29
Total (N = 36)					
	Experienced^**^ (N = 12)	2.52	1.36	3.45	2.00‐3.47
	Novice^**^ (N = 24)	6.55	6.63	5.37	4.05‐10.15

Abbreviations: CI, confidence interval of the median; IQR, interquartile range.

^*^ *P* = .0013.

^**^ *P* = .0012.

## 4 DISCUSSION

We described use of the Brainsight Vet 2 System with the equine skull using fiducial arrays or anatomic landmarks, with no significant difference between registration methods. When considering only the data from the experienced surgeon, accuracy when using either fiducial arrays (2.47 mm) or anatomic (2.58 mm) points of registration approximated the accuracy of other studies validating the Brainsight navigation system in healthy sheep (1.85 mm)^21^ and in dogs with naturally occurring brain tumors (1.79 mm).^1^, ^21^ Without consideration for level of experience, the overall accuracy in our study with anatomic landmarks (3.76 mm) and the fiducial array (5.75 mm) was lower than the previous studies using the same system,^1^ but similar to the application accuracy reported in a study using other optical navigation systems (3.6 mm),^3^ as well as application and needle accuracy reported using nonguided stereotactic biopsy systems (2.9 mm and 3.5 mm, respectively).^6^, ^7^ In previously reported studies using the Brainsight system, MRI‐guided brain biopsy in the dog with the standard fiducial arrays resulted in a mean distance from intended intracranial targets of 1.79 mm (SD = 0.87 mm)^1^ and MRI‐guided brainstem biopsy in sheep in a research setting resulted in a needle placement error of 1.85 ± 1.22 mm.^21^ Two other studies also evaluated accuracy of the Brainsight navigation system in various applications.^4^, ^24^ One study evaluated accuracy for placement of deep brain stimulation electrodes in dogs, for which accuracy was 4.6 mm (SD = 1.5 mm).^4^ The other study evaluated the accuracy of the Brainsight navigation system for radiation planning using a phantom dog skull, and found an accuracy of 1.3 mm (SD = 1.242 mm).^24^ Accuracy of frame‐based or frameless stereotactic brain biopsy systems for dogs in other studies was 3.6, 2.9, 3.5, and 1.5 mm.^3^, ^6^, ^7^, ^9^ Some of these systems, however, are for needle biopsy only,^6^, ^7^ and cannot be used for surgical navigation during mass resections. The Brainsight neuronavigation system is designed for either needle biopsy or real‐time guidance during mass resection.

The registration accuracy of anatomic landmarks in our study also was consistent with studies in humans evaluating the use of anatomic landmarks alone^14^ or in conjunction with fiducial arrays to further enhance accuracy.^13^ Optical neuronavigation using both of these registration methods was performed successfully by both clinicians in our study, with and without previous experience using optical neuronavigation systems. The feasibility of using anatomic registration points has substantial clinical impact, because the navigation planning using anatomic landmarks and intracranial surgical procedures can be performed during a single anesthesia event, thus eliminating the need for either a separate planning session or an additional procedure to surgically place the fiducial array, thereby minimizing morbidity and mortality risks associated with an additional anesthetic procedure.^10^, ^11^, ^12^ In a recent confidential enquiry into perioperative fatalities in horses (the CEPEF‐4 study), the 7‐day mortality rate for horses undergoing anesthesia, with death or euthanasia as a result of anesthetic recovery and not related to the primary disease, was 0.9%.^11^, ^12^ An additional 1% of horses had nonfatal anesthetic complications upon recovery.^12^ These results make the application of neuronavigation in horses clinically relevant. No prior procedures are required to use anatomic registration points. The standard fiducial array can be used during a single anesthesia event, but does require a minor surgical procedure for placement into the frontal bone. This array is secure once attached, but can be dislodged. While the horse is moved from the surgical area (to place the fiducial array) to the imaging area and back to the surgical area for the biopsy procedure, the array should be protected to avoid screw dislodgement. Should the clinician need to recover the patient and perform navigation in a separate anesthesia event, the array can be detached from the base plate by a small locking screw, and the patient's skin can be closed over the base plate. Once the navigation procedure commences, the skin incision can be reopened and the array reattached to the base plate. This approach is not necessary if the procedures are performed under a single anesthetic event and, in our study, the array was left in place throughout the entire procedure.

As with many highly technical procedures, surgeon experience plays a role in the accuracy of neuronavigation procedures. As expected, the surgeon experienced with neuronavigation systems was significantly more accurate than the novice surgeons when using the fiducial arrays. The lack of significance when comparing surgeon experience using the anatomic landmarks is likely because even the experienced surgeon had no prior experience using natural landmarks for registration in neuronavigation procedures, as it represented a novel adaptation to the standard procedures. Despite statistical significance, the mean distance from the seed tip to the intended target was smaller for both methods for the experienced surgeon. A study of physician neurosurgeons that evaluated the learning curve of standard and advanced neuronavigation instrumentation procedures indicated that the majority of experience‐related errors occurred in the first month, after which time there was no significant difference in accuracy between the novel and standard techniques.^25^ We were unable to detect sequential improvement for either surgeon during the course of our study, which is not surprising given the limited size of the study and the complexity of the procedure. Studies evaluating the learning curve for neuronavigation in veterinary species have not been done, but would be necessary to determine if the learning curve in veterinary neurosurgery is comparable to that reported for physician neurosurgeons.

The higher SD using anatomic landmarks may make fiducial arrays a more accurate option for small brain lesions, although in some studies of humans the combination of fiducial arrays and anatomic landmarks was even more accurate than fiducial arrays alone.^13^ Because of the presumed safety benefit of avoiding an additional anesthesia and recovery, and despite the increased variation, the use of anatomic landmarks remains a viable option in terms of overall accuracy and reduction of patient risk.

Determining a specific minimum brain lesion size applicable for using the Brainsight system for clinical navigation in horses was beyond the scope of our study. Such determinations typically are not extrapolated from accuracy data. Furthermore, the clinically relevant outcomes of brain biopsy are safety and diagnostic yield, both of which are multifactorial. Given the use of cadavers in our study, subsequent studies would be required to evaluate safety and diagnostic yield in clinically affected horses, but several studies have reported diagnostic yield in clinical canine patients.^8^, ^9^, ^26^, ^27^ One study using a modified Pelorus Mark III system, for which accuracy is reported as 3.5 mm, found a 91% diagnostic yield.^8^ Diagnostic yield was 96% when using another stereotactic biopsy system for which needle placement error was 2.9 mm.^26^ Another biopsy system for which median needle placement error was 1.5 mm resulted in a diagnostic yield of 87% in 1 study, but lesion volume was not statistically significant as a risk factor for diagnostic yield, and only needle placement error was considered a risk factor.^9^ An additional study using this same system resulted in a diagnostic accuracy of 100% in meningioma cases, and 81% in glioma cases.^27^ In that study, risk factors for discordance in biopsy diagnosis (eg, tumor grade) were evaluated for both gliomas and meningiomas, but risk factors were not reported for diagnostic versus nondiagnostic samples, specifically. Statistically significant risk factors for discordance in tumor grade included lesion volume, number of biopsies attempted, and number of biopsy samples obtained.^27^ The relative influence of these factors is not known.

Our study was limited by the use of cadaver heads, and further analysis in live patients is warranted. Decapitation and freeze‐thaw effects on the cadaver head include brain shift, pneumocephalus, and distortion of some anatomic landmarks. Once cadaver heads were thawed and prepared for use, the procedures and all imaging (pre‐ and postprocedure) occurred in a single event. Furthermore, the procedures using both methods of registration and biopsy (seed implantation) were performed during the same event, and on the same cadaver head, thus minimizing any variability these effects would have on the results. Furthermore, the order of performing the 2 methods (anatomic versus fiducial array) and the order of targeting the 6 biopsy sites in the equine cadaver head were randomly determined to avoid bias. Specific factors should be considered when interpreting our data. Given that brain shift may have occurred with the change in position between the pre‐ and post‐procedure imaging (which occurred with the cadaver heads in dorsal recumbency) and the biopsy procedure (in which the heads were positioned as if the horse was standing), it is best to consider our differences in actual versus intended target locations as relative accuracies between registration techniques, and not an empirical accuracy measurement. Although we did not identify noticeable shifting of soft tissue brain structures during our pre‐ and postprocedure image coregistration, the position of the brain could have shifted during the biopsy procedure, because of the change in patient orientation. Additionally, as a result of the freeze‐thaw cycle, the left eye of 1 cadaver head was distorted and could not be used as an anatomic landmark but this amended registration protocol still exceeded the minimum number of landmarks necessary for optical navigation using the Brainsight Vet system. Despite these variations in registration protocols, and despite the use of cadavers in our study, we found consistent accuracy between the 2 methods of registration.

It is also possible that coregistration of pre‐ and postprocedural images could have introduced additional minor errors, either in a positive or negative manner, although such errors are expected to be minor because CT images have high spatial resolution and rigid, bony structures were used for coregistration in multiple planes.

Although not yet commonplace, because of improved availability of multiplanar imaging in horses and increased interest by horse owners to diagnose and treat intracranial diseases, the Brainsight optical neuronavigation system likely will become an increasingly important surgical tool for the equine patient. Neuronavigation‐guided intracranial procedures have been thoroughly studied in dogs and cats,^1^, ^2^, ^3^, ^4^, ^24^, ^28^ as well as in nonhuman primates and sheep,^15^, ^16^, ^17^, ^18^, ^19^, ^20^, ^21^ but have not yet been described in the horse. The validation of neuronavigation in horses provides a basis for which definitive diagnoses by biopsy of masses such as ependymomas,^29^ hamartomas,^30^ abscesses,^23^ and granulomas^22^, ^31^, ^32^, ^33^, ^34^, ^35^ may be achieved antemortem. Additionally, cerebral masses or adenomas of the pituitary pars intermedia may be resected using minimally invasive neuronavigation‐guided surgical techniques. Improved accuracy in these intracranial procedures is important to minimize morbidity in these large animals.

In conclusion, optical neuronavigation using the Brainsight Vet 2 system is feasible in horses and, based on our preliminary data, the use of anatomic landmarks appears to be a feasible option for registration. Optical neuronavigation should be considered when performing brain biopsies or surgical procedures in horses that may require a high degree of accuracy, such as surgical treatment of Cushing's disease and biopsy or resection of inflammatory lesions, granulomas, or other masses, and may allow such procedures to become more feasible. Surgeon experience impacts accuracy, and highlights the need for further studies to evaluate the appropriate level of training needed for performance of these procedures.

## CONFLICT OF INTEREST DECLARATION

Authors declare no conflict of interest.

## OFF‐LABEL ANTIMICROBIAL DECLARATION

Authors declare no off‐label use of antimicrobials.

## INSTITUTIONAL ANIMAL CARE AND USE COMMITTEE (IACUC) OR OTHER APPROVAL DECLARATION

All activities were performed in accordance with appropriate IACUC and CRB approvals or exemptions.

## HUMAN ETHICS APPROVAL DECLARATION

Authors declare human ethics approval was not needed for this study.

## Supporting information


**Table S1** Anatomic landmarks used for each specimen. Note that after Horses 1 and 2, the infraorbital foramina were excluded due to inconsistency and lack of repeatability, and the study design was adapted to use the tip of the facial crest in lieu of the infraorbital foramina for remaining horses. The left eye of Horse 3 was distorted and had to be omitted from registration in that case. Random number charts were used to determine the procedural order for the anatomic landmarks or fiducial array.
