# Supplementary material for: A pilot study of optical neuronavigation‐guided brain biopsy in the horse using anatomic landmarks and fiducial arrays for patient registration
Source: J Vet Intern Med. 2020 May 29;34(4):1642–9. doi: 10.1111/jvim.15813 (PMC7379038; doi:10.1111/jvim.15813)
Supplement: Supplementary file 1 — Table S1 Anatomic landmarks used for each specimen. Note that after Horses 1 and 2, the infraorbital foramina were excluded due to inconsistency and lack of repeatability, and the study design was adapted to use the tip of the facial crest in lieu of the infraorbital foramina for remaining horses. The left eye of Horse 3 was distorted and had to be omitted from registration in that case. Random number charts were used to determine the procedural order for the anatomic landmarks or fiducial array. [file JVIM-34-1642-s001.pdf]

Supplemental Table 1. Anatomic landmarks used for each specimen. Note that after Horses 1 and 2, the infraorbital foramina were excluded due to inconsistency and lack of repeatability, and the study design was adapted to use the tip of the facial crest in lieu of the infraorbital foramina for remaining horses. The left eye of Horse 3 was distorted and had to be omitted from registration in that case. Random number charts were used to determine the procedural order for the anatomic landmarks or fiducial array.

Specimen	Anatomic landmarks
Horse 1	Right and left medial canthus, right and left lateral canthus, left and right infraorbital foramen, left and right supraorbital foramen, bregma
Horse 2	Right and left medial canthus, right and left lateral canthus, left and right infraorbital foramen, left and right supraorbital foramen, bregma
Horse 3	Right and left medial canthus, right and left lateral canthus, left and right facial crest, left and right supraorbital foramen, bregma
Horse 4	Right medial canthus, right lateral canthus, left and right facial crest, left and right supraorbital foramen, bregma
Horse 5	Right and left medial canthus, right and left lateral canthus, left and right facial crest, left and right supraorbital foramen, bregma
Horse 6	Right and left medial canthus, right and left lateral canthus, left and right facial crest, left and right supraorbital foramen, bregma
